# A STING agonist preconditions against ischaemic stroke via an adaptive antiviral Type 1 interferon response

**DOI:** 10.1093/braincomms/fcac133

**Published:** 2022-05-24

**Authors:** Nandini Kundu, Amit Kumar, Carlo Corona, Yingxin Chen, Sonia Seth, Saravanan S. Karuppagounder, Rajiv R. Ratan

**Affiliations:** Burke Neurological Institute and Brain and Mind Research Institute, Weill Cornell Medicine, 785 Mamaroneck Ave, White Plains, NY, USA; Burke Neurological Institute and Brain and Mind Research Institute, Weill Cornell Medicine, 785 Mamaroneck Ave, White Plains, NY, USA; Burke Neurological Institute and Brain and Mind Research Institute, Weill Cornell Medicine, 785 Mamaroneck Ave, White Plains, NY, USA; Burke Neurological Institute and Brain and Mind Research Institute, Weill Cornell Medicine, 785 Mamaroneck Ave, White Plains, NY, USA; Burke Neurological Institute and Brain and Mind Research Institute, Weill Cornell Medicine, 785 Mamaroneck Ave, White Plains, NY, USA; Burke Neurological Institute and Brain and Mind Research Institute, Weill Cornell Medicine, 785 Mamaroneck Ave, White Plains, NY, USA; Burke Neurological Institute and Brain and Mind Research Institute, Weill Cornell Medicine, 785 Mamaroneck Ave, White Plains, NY, USA

**Keywords:** STING agonist, tilorone, TBK1, stroke

## Abstract

Converging lines of inquiry have highlighted the importance of the Type I antiviral response not only in defending against viruses but also in preconditioning the brain against ischaemic stroke. Despite this understanding, treatments that foster brain resilience by driving antiviral interferon responses have yet to be developed for human use. Studies from our laboratory showed that tilorone, the first human antiviral immunomodulatory agent to be developed, robustly preconditioned against stroke in mice and rats. Tilorone is a DNA intercalator; therefore, we hypothesized that it stabilizes cytosolic DNA (released from the mitochondria or the nucleus), thereby activating cyclic GMP–AMP synthase, a homeostatic DNA sensor, and its downstream pathway. This pathway involves ***st***imulator of ***in****t*erferon ***g***enes (STING), tank-binding kinase 1 (TBK1), and ***i***nterferon ***r***egulatory ***p***rotein-3 and culminates in a protective Type I interferon response. We tested this hypothesis by examining the ability of structurally diverse small-molecule agonists of STING to protect against oxygen/glucose deprivation *in vitro* in mouse cortical cultures and *in vivo* against transient ischaemia in mice. The STING agonists significantly reduced cell death both *in vitro* and *in vivo but* failed to do so in STING knockout mice. As expected, STING agonist-induced protection was associated with the induction of interferon related genes and the effects could be abrogated *in vitro* by a TBK1 inhibitor. Taken together, these findings in mice identify STING as a therapeutic target for preconditioning the brain against ischaemic stroke *in vitro* and *in vivo*. Moreover, they suggest that clinically approved STING agonists such as Ganciclovir or α-Mangostin are candidate drugs that could be tested in humans as a prophylactic treatment to alleviate brain injury associated with ischaemic stroke.

## Teaser

Kundu et al. report that 5,6-dimethylxanthenone-4-acetic acid (DMXAA) can activate *st*imulator of *in*terferon *g*enes (STING), triggering interferon-associated gene expression both *in vitro* and *in vivo*. They show DMXAA’s STING-dependent, protective, preconditioning effects in an ischaemic stroke model, suggesting STING as a potential therapeutic target for prophylaxis against stroke.

## Graphical Abstract

**Graphical Abstract:**
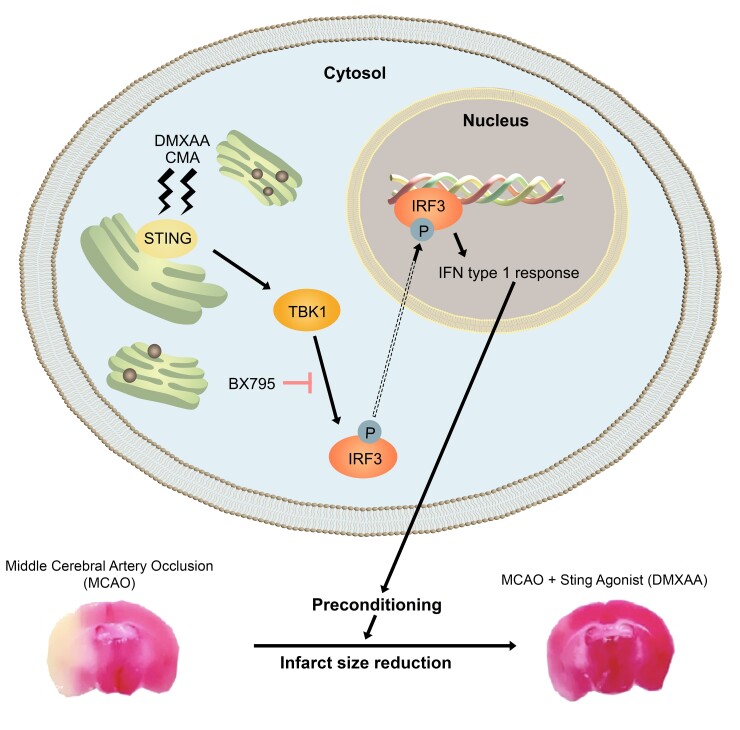

## Introduction

Ischaemic preconditioning describes the phenomenon wherein a sublethal ischaemic stimulus induces durable protection against a subsequent lethal ischaemic stimulus.^1,2^ In brain research, this preconditioning can be exploited to probe the pathways that are available to the body to protect against the consequences of lethal ischaemia, also known as stroke.^3–5^ Given the high predictability of stroke following cardiac bypass or aneurysmal coiling,^6^ an urgent need exists for agents that are capable of inducing preconditioning as a potential prophylactic treatment. Despite the obvious and immediate biological and therapeutic impact of preconditioning, agents that can precondition the brain in humans have yet to be developed.

The present knowledge of preconditioning agents has come from studies of innate immunity, which have provided a detailed molecular understanding of the convergent pathways induced by these agents.^4,5,7,8^ The innate immune system has evolved sensors, known as Toll-like receptors (TLRs), which are capable of sensing components of invading pathogens as well as substances released during sterile injuries. Once an infection or sterile injury is sensed by high-affinity binding of ligands to TLRs, the activated TLRs can foster cell damage and death. In contrast to acute infection, the same ligand-mediated activation of TLRs in advance of an injury can reprogramme the response to ischaemia and lead to neuroprotection.^5,9–12^ Indeed, bacterial LPS (Lipopolysaccharide), poly I:C (polyinosinic:polycytidylic acid, similar to double stranded RNA in some viruses) and ischaemia itself can all activate TLRs to precondition the brain against a subsequent lethal stroke.^13,14^

Elegant transcriptomic studies of RNAs from brains preconditioned with LPS, poly I:C, and ischaemia have defined a common signature of 13 genes that are activated by distinct preconditioning stimuli.^14^ Unbiased analyses of the promoter region for these genes have demonstrated enrichment of interferon response elements (IREs). Two transcription factors, interferon regulatory factor-3, and interferon regulatory factor-7, that can bind to IREs are involved in the induction of interferons and co-regulated genes. As expected from these findings, germline deletion of these transcription factors abrogates the preconditioning normally induced by these diverse stimuli.

The ability of interferon regulatory factors 3 and 7 to drive preconditioning in the brain has focused attention on the signalling pathways downstream of the TLRs that are capable of activating these factors. The hope is that these signalling pathways will provide additional druggable targets for preconditioning-induced brain protection. Indeed, using an unbiased drug screen for activators of the hypoxic adaptive response, we were able to identify the first antiviral interferon-inducing agent, tilorone, as our best hit.^15,16^

Tilorone is currently only used in humans in Europe and Russia as an antiviral agent;^17,18^ however, our studies showed that tilorone could robustly precondition against ischaemia in rats and mice.^19^ More importantly, its protective effects were associated with a significant induction, in multiple organs of the body, of a subset of the 13 genes (e.g. *Ifit1*, *Irf3*, *Irf7*) common to other preconditioning stimuli. Indeed, the forced expression of one of these genes, *Ifit1*, inhibited cell death induced by oxygen and glucose deprivation (OGD).

Tilorone is a DNA intercalator, so we hypothesized that tilorone induces interferon production by intercalating into and stabilizing cytosolic DNA (released following injury from mitochondria or the nucleus).^15^ Increased amounts of stabilized cytosolic DNA trigger the activation of a DNA sensor, cyclic GMP–AMP synthetase (cGAS).^20^ This cGAS activation produces cAMP-GMP, which is an agonist for the endoplasmic reticulum adaptor protein, stimulator of interferon genes (STING). Activated STING recruits tank-binding kinase I (TBK1), which ultimately leads to the phosphorylation and activation of IRF3.^21–27^

 Tilorone is not approved in the US due to concerns that it can cause mucopolysaccharidosis in rodent models. Therefore, we sought to evaluate the STING-TBK1-IRF3 pathway as a target for preconditioning against ischaemic stroke. In this study, we show that STING is a target, both *in vitro* and *in vivo*, for preconditioning against ischaemia. Recent studies have elucidated FDA-approved STING agonists;^28^ therefore, our studies should stimulate a new focus on STING activation as a strategy to induce ischaemic preconditioning in humans.

## Materials and methods

Animals: Experiments and procedures on mice were approved by the Institutional Animal Care and Use Committee of Weill Cornell Medical College and were in accordance with the guidelines established by the National Institutes of Health and Animal Research: Reporting of *In Vivo* Experiments. All mice were housed in a pathogen-free facility on a 12-h light/dark cycle from 6am to 6pm and provided ad libitum access to food and water. Animals used in *in vivo* studies were 8–10-week-old male C57BL/6 mice (Charles River) and STING KO mice (Strain #017537; Jackson Laboratories).

### Mature neuronal cell culture and viability

Primary neuronal cultures were obtained from embryonic day 15 pups (mixed gender) from 8–12-week-old pregnant female CD1 mice (Charles River) as described in.^29^ Mature neurons were cultured (days *in vitro* 15) as previously described in.^19^ Neurons were identified by visual morphology. Mature neurons were sensitive to OGD-induced cell death and were completely inhibited by MK801 (an ionotropic glutamate receptor antagonist; Sigma Aldrich; 10μM) treatment.

The mature neurons were treated with different doses of 5,6-dimethylxanthenone-4-acetic acid (DMXAA) (Sigma Aldrich) dissolved in varying concentrations of DMSO/media. DMXAA or 10-carboxymethyl-9-acridanone (CMA) (a Sting agonist structurally diverse from DMXAA, Tocris) were added with or without BX795 (a TBK1 inhibitor; Sigma Aldrich) for 12 h (preconditioning). After washing three times with glucose-free media, the cells were replaced with glucose-free media and transferred to a hypoxia chamber in OGD conditions for 3.5 h. A parallel plate in normal media was incubated in an incubator (normoxia). OGD was terminated by replacement with neurobasal media. The cultures were returned to the incubator for 24 h and then cell viability was assessed by LIVE staining with membrane-permeant, calcein AM. Calcein positive neurons (green fluorescence) were quantitated in a blinded fashion using blinded cell counting. MK801 (Sigma Aldrich; 10μM) was used to verify that OGD-induced death depended on ionotropic glutamate receptor activation.

### Murine model of middle cerebral artery occlusion

Middle cerebral artery occlusion (MCAO) surgeries were conducted as previously described.^19,30^ All surgeries were conducted under aseptic conditions by a skilled, mouse surgeon. Male C57BL/6 mice (8–10-weeks-old, weight ranging from 24–29 g) or STING KO were anaesthetized with isoflurane (5% induction and 2% maintenance). A 2 cm incision was opened in the middle of the anterior neck. The right common carotid was temporarily ligated with 6-0 silk (Ethicon Inc.). Right unilateral MCAO was accomplished by inserting a silicon rubber-coated monofilament (Doccol Corporation) into the internal carotid artery via the external carotid artery stump. Body temperature was controlled at 36.5 ± 0.5°C throughout MCAO surgery with warm water pads and a heating lamp. After 45 min of occlusion, the occluding filament was withdrawn to allow for reperfusion and the incision was closed with 6-0 surgical sutures (ETHICON, Inc). 0.5 mL prewarmed normal saline was given subcutaneously to each mouse after surgery. Mice were then allowed to recover from anaesthesia and survived for 24 h after initiation of reperfusion. The surgeon was blinded to treatment groups. Mice were euthanized, and brains were collected at 24 h of reperfusion for 2,3,5-triphenyltetrazolium chloride (TTC) (Sigma, St. Louis, MO) staining. Treatment groups received DMXAA (25 mg/kg) dissolved in vehicle (DMSO:PEG:β-cyclodextrin at a ratio of 5:45:50).^31^

### Infarct volume analysis

Infarct volume measurements were conducted as previously reported.^30^ Animals were euthanized, and brains were harvested 24 h hours after reperfusion for TTC (Sigma, St. Louis, MO) staining to determine the volume of cerebral infarction. The brain sections with 2 mm thickness were incubated in 12% TTC solution at 37°C for 15 min. Then, the tissue sections were removed and fixed in formalin solutions (10%) for 24 h. The digital imaging was used to analyze the Infarction volume and Sigma Scan Pro 5.0 Software (Systat, Inc, Point Richmond, CA) was used to process the images. Brain oedema was corrected by subtracting the ipsilateral non-infarcted regional volume from the contralateral regional volume to determine infarct volume. Thereafter, obtained value was divided by the contralateral regional volume and multiplied by 100 to get regional infarction volume as a percent of the contralateral regional volume. The experimenter analyzing the infarct volume was blinded to the vehicle and treatment group.

### Quantitative real-time polymerase chain reaction

Quantitative real-time polymerase chain reaction (RT-qPCR) analysis were carried out as previously described.^30^ NucleoSpin RNA II kit (Clonetech) was used to extract RNA from either cell or tissue samples using the manufacturer’s protocol. To quantify genes of interest, gene expression assays with FAM–labelled probes (Thermo Scientific) were used in Duplex real-time PCR reactions. *Ifit1* (Mm00515153) primer was normalized withβ-actin labelled with VIC–labelled probe. Master mix for sample mRNA were prepared with TaqMan RNA to Ct 1-step kit (Applied Biosystems) and 7500 Real-Time PCR System (Applied Biosystems) was used to conduct all experiments in accordance with the 1-step qPCR protocol (Applied Biosystems). Mean and SD was calculated from a minimum of 3–4 independent experiments from qPCR.

### RT2 profiler polymerase chain reaction array analysis

Primary neurons were isolated and cultured from E14 mice embryos of wild-type (WT) and STING knockout mice using the method as previously described.^30^ RNA was extracted and quantified as using a NucleoSpin RNA II Kit (Clontech, catalog number—740955-250). cDNA was generated using RT^2^ First strand kit (Qiagen, catalog number—330401) and RT^2^ SYBR Green qPCR Mastermix (Qiagen, catalog number—330503). cDNA was used in mouse interferons and receptors 96 wells array plates (Qiagen, catalog number—PAMM-064ZC) for performing RT^2^ Profiler PCR Array. Raw data were analyzed using Qiagen gene globe web portal (http://www.qiagen.com/geneglobe). GraphPad Prism 9.3 was used to generate the heat map.

### Image analyses

For quantification, digital images were processed using Image J software developed at the National Institute of Health, Bethesda, MD. Three to five images were quantified for each condition and performed triplicates for each study. We used automated counting of single-colour images by converting them to greyscale, followed by background subtraction and binary images of the analyzed particles (pixel size; 100) were counted. Total cells in the controls were considered as 100%, and rest other conditions were compared with it. The analysis was performed in a blinded fashion.

### Statistical analyses

Data are reported as means ± standard deviation of multiple individual experiments. The statistical analyses were carried out with Microsoft Excel and GraphPad Prism. A two-tailed *t*-test was used if two groups were compared, a one-way ANOVA with Dunnett’s multiple comparisons or test if more than two groups were compared. We also used repeated measures one-way ANOVA with Dunnett’s multiple comparisons test, repeated measures two-way ANOVA with Tukey’s multiple comparison and two-way ANOVA with Šídák’s multiple comparisons test for statistical analyses.

## Data availability

The data that support the findings of this manuscript are available from the corresponding authors upon reasonable request.

## Results

### Stimulator of interferon genes agonists induce a stereotypical interferon response in mature murine neuronal cultures

Previous studies from our laboratory showed that tilorone, as an interferon inducer and DNA intercalator, can precondition the brain against stroke.^16,19^ These findings are congruent with a model in which brain preconditioning by tilorone occurs via stabilization and accumulation of cytosolic DNA. This accumulation leads to activation of the ER transmembrane protein, STING, subsequent recruitment of TBK1, and phosphorylation of IRF3. The phospho-IRF3 can bind with high affinity to the promoters of interferon-associated genes to drive the induction of their transcription.

**Figure 1:**
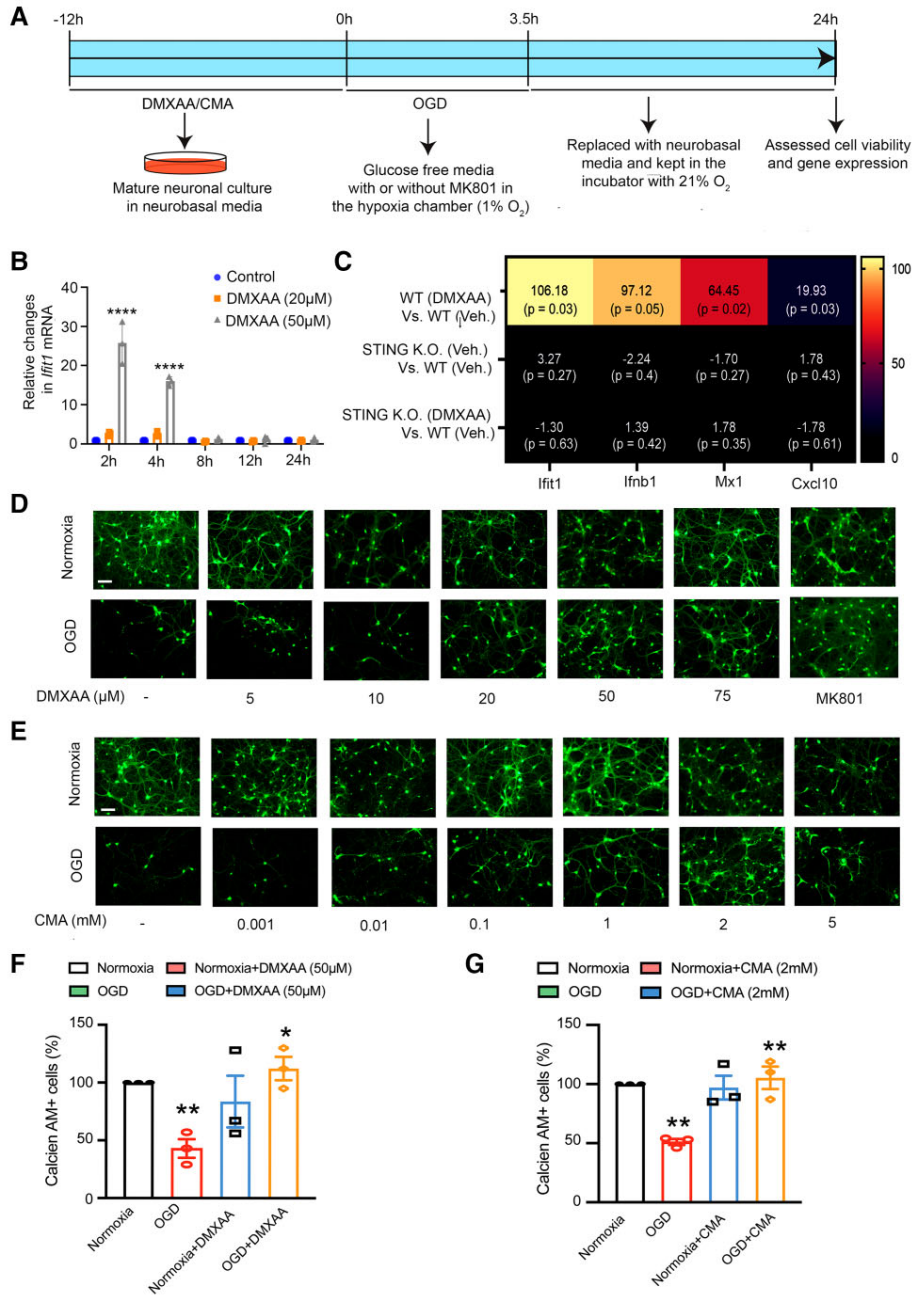
**Preconditioning with the canonical STING agonists, DMXAA or CMA drives antiviral IFN stress response gene expression in primary neuronal cultures and protects against OGD-induced death.** (*A*) Experimental design of *in vitro* experiments shown in B-G. (*B*) qPCR analysis of *Ifit1* gene over time in mature neurons. The statistical analysis was performed using repeated measures Two-way ANOVA with Tukey’s multiple comparisons test. Data are expressed as mean ± standard deviation of three independent biological replicates (*n* = 3). ****P < 0.0001 compared with the control group. The analysis revealed significant effects for time (F_(4, 30)_ = 70.94, *P* < 0.0001), treatment (F_(2, 30)_ = 144.2, *P* < 0.0001), and interaction (F_(8, 30)_ = 55.51, *P* < 0.0001). (*C*) Heat map representation of relative changes in gene expression of *Ifit1*, *Ifnβ1*, *Mx1,* and *Cxcl10* genes from the gene array study. The total RNA for the study was extracted in samples from WT and STING KO mouse primary neurons treated with DMXAA for 2 h. The statistical analysis of the gene array data was performed using Qiagen automated analysis web platform (http://www.qiagen.com/geneglobe) and individual *P* values were obtained from the excel sheet generated from the analysis. (*n* = 3) (*D, E*) Representative live images of STING agonists preconditioning, followed by 3.5 h of OGD condition, followed by 24 h reperfusion in mature neurons with different concentration of structurally different STING agonists, DMXAA (*D*) and CMA (*E*). 50 µM DMXAA (*D*) and 2 mM CMA (*E*) preconditioning provided neuroprotection against OGD in mature mouse neurons. As a positive control, MK-801 showed protection in OGD-induced death in mature neurons (*D*). (*F, G*) Image quantification using ImageJ software of 50 µM DMXAA (*D*) and 2 mM CMA (*E*). Each data point in (*F*) and (*G*) represents an independent set of experiment. Scale bar, 50 µm.

The first prediction of this model is that small-molecule chemical agonists of STING should not only drive expression of interferon-associated genes but should also precondition against cell death in response to ischaemia *in vitro* and *in vivo*. We tested this prediction by exposing mature primary neuronal cultures (composed of neurons and glia) to the selective STING agonist, 5,6-dimethylxantheonone-4-acetic acid (DMXAA). As expected, a 2 h exposure to 50 μM DMXAA resulted in a significant increase in *Ifit1*. (Fig. 1B). Next, to verify that DMXAA effects are attributable to STING, and not some off-target effect, we examined the effect of DMXAA in wild-type (WT) and STING KO neurons. In WT neurons, DMXAA significantly induced *Ifit1* and other interferon gene signatures including interferon beta 1 (*Ifnβ1*), MX Dynamin Like GTPase 1 (*Mx1*) and C-X-C Motif Chemokine Ligand 10 (*Cxcl10*) included in the Type I Interferon array. As expected, upregulation of these genes (*Ifit1*, *Ifnβ1*, *Mx1,* and *Cxcl10*) was significantly abrogated in STING KO neurons (Fig. 1C). Taken together, these data suggest that DMXAA can act on neurons or glia to activate STING and induce interferon-associated genes.

### Preconditioning with a stimulator of interferon gene agonist provides neuroprotection against ischaemic injury *in vitro*

Our observation of a STING-dependent induction of interferon genes in mature neurons led us to ask if preconditioning with DMXAA would provide neuroprotection against ischaemia *in vitro*. We modelled ischaemic stroke *in vitro* by exposing mature neurons to 3.5 h of OGD, followed by 24 h of normoxia and normoglycaemia (reperfusion) (Fig. 1A). As expected, 24 h after the 3.5 h OGD treatment, the majority of the mature neurons had died, as determined by reductions in live-cell staining (Calcein AM, Fig. 1D and E) or LDH release (not shown). MK-801, a non-competitive NMDA receptor antagonist, prevents OGD-induced death, consistent with a significant excitotoxic component to this death (Fig. 1D and E).

We then tested whether treatment with a STING agonist would precondition neurons against OGD-induced cell death. We treated mature neurons with various doses of DMXAA for 12 h prior to a 3.5 h exposure to OGD and 24 h of reperfusion. Consistent with our model, we observed that preconditioning with 50 μM DMXAA imparted resistance to OGD-reperfusion injury *in vitro* (Fig. 1D and F).

We then verified that the effect of DMXAA was “on target” as a STING agonist by examining the ability of a structurally distinct, murine STING agonist, CMA, to prophylax against OGD-induced death. As expected, pretreatment with 2 mM CMA also protected against OGD-induced death (Fig. 1E and G). These findings suggested that DMXAA and CMA are working via their common target, STING, to precondition against ischaemic injury *in vitro*.

### Stimulator of interferon gene agonists are acting via a canonical tank-binding kinase 1 pathway to precondition against ischaemia *in vitro*

**Figure 2:**
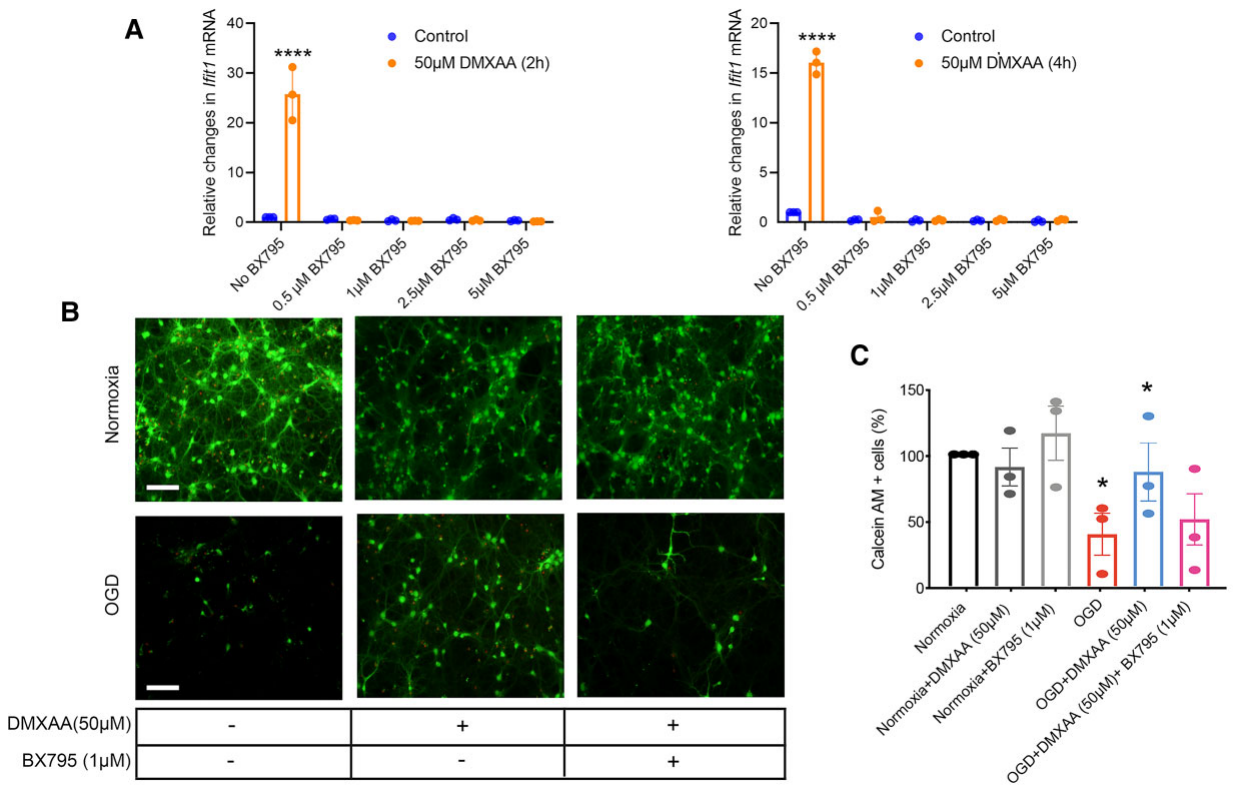
**BX795 (an inhibitor of TBK1, a component of the STING pathway) inhibits STING agonist-mediated upregulation of *Ifit1* and is correlated with the abrogation of neuroprotection with a STING agonist.** (*A*) qPCR analysis of *Ifit1* gene expression with 50 µM DMXAA and different doses of BX795 for 2 and 4 h. The statistical analysis was performed using Two-way ANOVA with Šídák’s multiple comparisons test. Data are expressed as mean ± SD (*n* = 3). ****P < 0.0001 compared with the control group. (*B*) Representative live images of STING agonists preconditioning, followed by 3.5 h of OGD condition, followed by 24 h reperfusion in mature neurons with 50 µM DMXAA and BX795 (1 µM). (*C*) Image quantification using ImageJ software of 50 µM DMXAA and 1 µM BX795. Each data point represents an independent set of experiment.

Activated STING recruits TBK1, and this complex of proteins is required for the TBK1-dependent phosphorylation of IRF-3.^27^ We verified the need for TBK1 in STING agonist-mediated neuroprotection by testing BX795, a selective inhibitor of TBK1. BX795 has been shown previously to block IRF3 activation and transcription but did not block NF-kappa B signalling in response to TLR3 or TLR4 agonists.^32^ Here, we monitored *Ifit1 levels by qPCR* to determine whether induction of IFN-associated genes by STING agonists would be blocked by BX795. We found clear pharmacological inhibition of TBK1 activity by BX795. Even at the lowest doses of BX795, we observed inhibition of *Ifit1* induction by DMXAA (Fig. 2A). As expected from these findings, BX795 also abrogated preconditioning by the STING agonist, DMXAA (Fig. 2B and C). Taken together, these data suggest that DMXAA (and CMA) precondition neurons against ischaemia *in vitro* via a canonical STING-TBK1-IRF3 pathway.

### The stimulator of interferon gene agonist DMXAA induces Type I interferon-associated expression in the mouse brain, liver, spleen, and kidney

**Figure 3:**
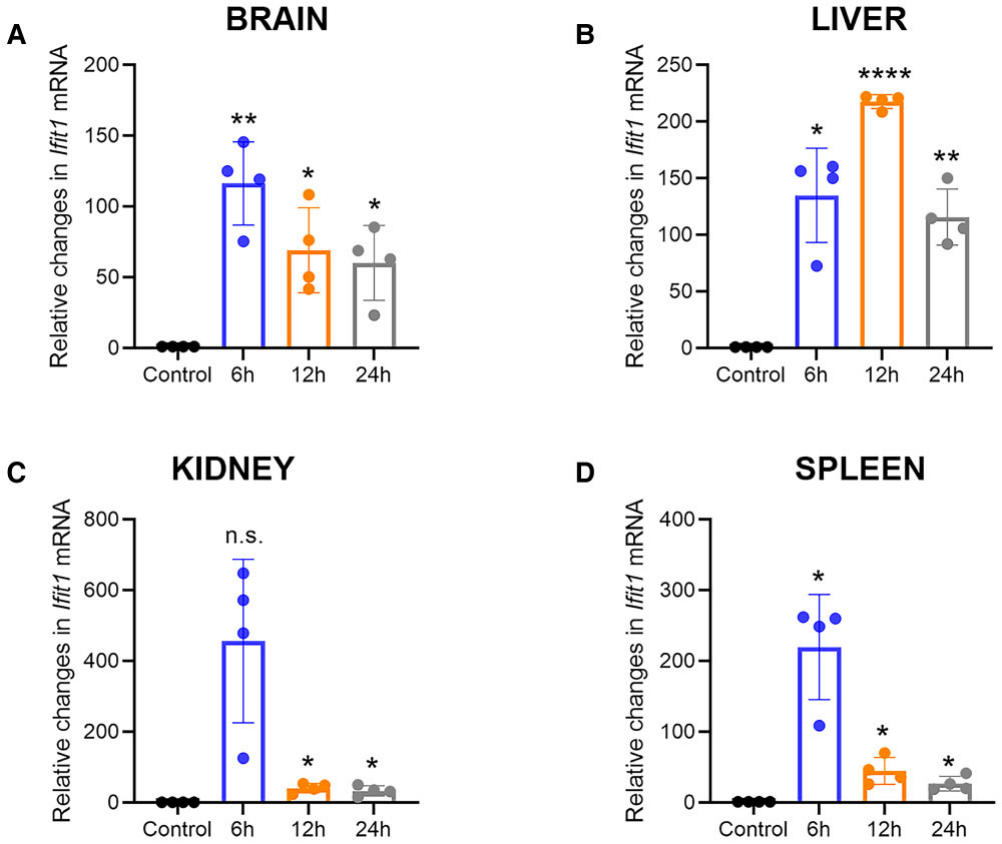
**DMXAA preconditioning increases antiviral interferon response gene expression *in vivo*. qPCR analysis of *Ifit1*(*A–D*) gene expression with 25 mg/kg DMXAA in a time-dependent manner, in mice brain, liver, spleen, and kidney.** The repeated measures one-way ANOVA with Dunnett's multiple comparisons test revealed significant effects for the time (Brain: F_(1.030, 3.091)_ = 11.96, *P* = 0.0387; Liver: F_(1.035, 3.105)_ = 42.77, *P* = 0.0065; Kidney: F_(1.003, 3.009)_ = 13.22, *P* = 0.0356; Spleen: F_(1.029, 3.008)_ = 23.71, *P* = 0.0154). Data are expressed as mean ± SD (*n* = 3). ****P < 0.0001, **P < 0.01, *P < 0.05 compared with the control group.

We determined whether STING activation could precondition the brain in living mice by testing the effects of DMXAA pretreatment on transient cerebral ischaemia. A 25 mg/kg dose of DMXAA, delivered intraperitoneally, induced the interferon-associated genes *Ifit1* (Fig. 3A–D). Subsequent qPCR analysis showed that *Ifit1* mRNA levels from brain lysates peaked at 6 h following DMXAA treatment but were sustained above vehicle-treated animals at 24 h (Fig. 3A). In the liver, the *Ifit1* levels peaked at 200-fold at 12 h following DMXAA injection, and, as in the brain, the levels at 24 h were sustained significantly above those of the control (Fig. 3B). The spleen also showed a similar pattern of induction to that seen in the brain and liver (Fig. 3C). Taken together, our data suggest that preconditioning with the STING agonist DMXAA is correlated with an expected significant, and temporally correlated, increase in *Ifit1*, an IFN-associated gene in the liver, spleen, brain, and kidney.

### Preconditioning with DMXAA in an middle cerebral artery occlusion model reduces infarct volume in mice

**Figure 4:**
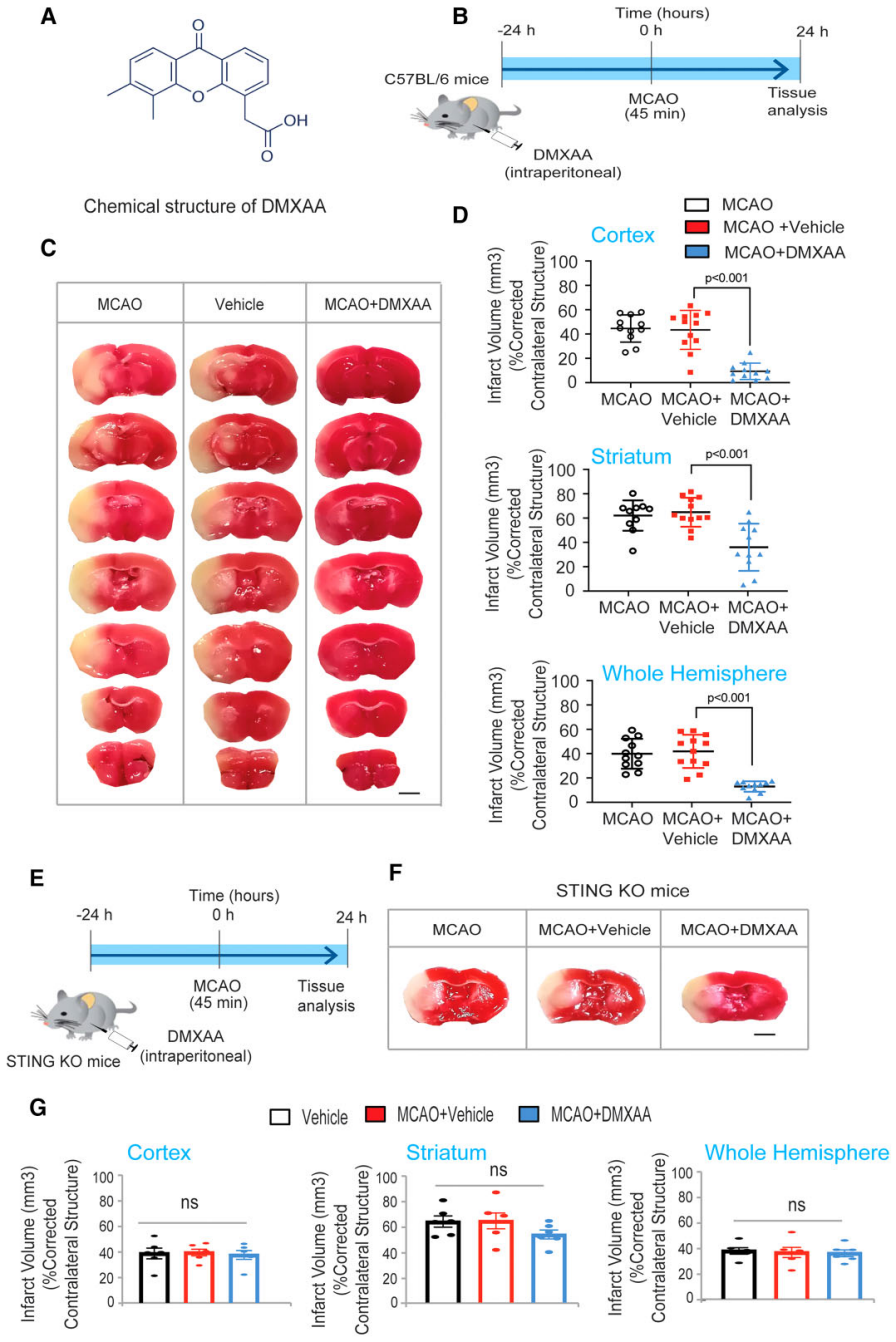
**Pretreatment with DMXAA can precondition against stroke-induced damage in mice.** (*A*) The chemical structure DMXAA. (*B*) DMXAA treatment and the MCAO model protocol. (*C*) Infarct volumes shown are the cortex (*P* < 0.05 for DMXAA compared with control), striatum and hemisphere infarct volumes. Data are expressed as mean ± SEM. A two-way ANOVA with Dunnett’s test was used to calculate the significance. All the treatments were randomized for *in vivo* and treatment group assignments. (*D*) Representative images of brain sections after TTC staining 24 h after transient MCAO. Preconditioning of 25 mg/kg DMXAA (*n* = 10 mice/group) significantly reduced infarct size 24 h after MCAO. (*E*) DMXAA treatment in STING KO mice. (*F*) Representative images of brain sections after TTC staining 24 h after transient MCAO. (*G*) Preconditioning of 25 mg/kg DMXAA (*n* = 5 mice/group) failed to reduce infarct size in STING KO mice 24 h after MCAO. Scale bar, 250 µm. Data are expressed as mean ± SEM. A two-way ANOVA with Dunnett’s test was used to calculate the significance.

We determined if STING activation preconditioned against ischaemic stroke by injecting mice intraperitoneally with the STING agonist DMXAA (25 mg/kg) 12 h prior to a 45 min treatment of MCAO (Fig. 4A and B). Systemic DMXAA exposure prior to MCAO resulted in significantly reduced cortical, striatal and total hemispheric infarct volumes at 24 h (Fig. 4C and D). This effect could not be attributed to DMXAA-induced changes in body weight or temperature. To verify that STING is the target for DMXAA-induced ischaemic protection, we tested the effect of DMXAA *in vivo* in STING KO mice. In contrast to the protective effects of DMXAA in WT mice, DMXAA preconditioning in STING KO had no effect on the MCAO induced ischaemic stroke (Fig. 4E–G). These data show that pretreatment with a STING agonist protects against a subsequent ischaemic stroke (Fig. 4C and D) and that this resilience is associated with a systemic Type I Interferon response. Together, these data define STING as a bona fide target for inducing tolerance to ischaemia in mice.

## Discussion

Innate immune signalling triggered by TLRs is known to mediate adaptive Type I IFN transcriptional responses that lead to resilience to stroke in mice, rats, and non-human primates.^4,5,7–12,14^ Recent studies from our lab have built on these important observations to demonstrate that tilorone, the first oral antiviral interferon-inducing agent, can precondition the brain against stroke.^19^ Tilorone is a DNA intercalator;^33–37^ therefore, we hypothesized that it triggers protective IFN responses by its ability to stabilize cytosolic DNA.

Accumulation of cytosolic DNA triggers a canonical antiviral response involving STING, TBK1, and IRF3, culminating in the induction of Type I interferons.^15^ Type I Interferons induce an antiviral state in all cells of the body via innate immunity and enforce B and T cells functions to optimize adaptive immunity. In this study, we used chemical and biological tools to show that STING activation is a viable strategy for preconditioning the brain against stroke. We used structurally diverse chemical activators of STING,^22,24,38^ as opposed to molecular activation because chemical tools allow the manipulation of STING on a more precise temporal scale, with fewer concerns about secondary changes that might emerge with molecular manipulations. Evidence that our chemical activators of STING are selective is supported by data showing induction of interferon-associated genes *in vitro* in cultured neurons is reduced significantly in the STING KO mice. As expected from these observations, preconditioning against rodent stroke by the STING agonist DMXAA is also reduced in the STING KO mice. Altogether these data support STING agonists as a strategy for inducing preconditioning in the mouse brain. The resilience to stroke we observed with STING agonists was also quantitatively similar to that induced by sublethal ischaemia, TLR agonists (LPS, poly I:C), and tilorone, which have also been shown to induce Type I Interferon signalling to precondition the brain.

We also provide evidence supporting a role for the kinase TBK1, which is recruited downstream of activated STING to trigger preconditioning via phosphorylation of IRF3. As expected from this model, neuroprotection *in vitro is abrogated* by a potent inhibitor of TBK1. Future studies will verify whether TBK1 is induced *in vivo* in immune cells, microglia, astrocytes and/or neurons *in vivo* to mediate STING-mediated neuroprotection.

Recent studies have identified the antiviral ganciclovir (GCV) as a STING agonist that can induce a protective Type I interferon response, reduce microglial reactivity and suppress inflammation in animal models of multiple sclerosis, independent of its thymidine kinase activity.^28^ Through the STING pathway, GCV reduces the microglial inflammatory signature in EAE models.^28^ During ischaemic stroke, microglia are activated and have been shown to release pro-inflammatory cytokines that induce further damage.^39^ These findings, in addition to those presented herein (Fig. 1) suggest that STING agonists such as GCV might precondition by reducing microglial activation in addition to fortifying neurons and astrocytes. These observations, along with GCV’s approval for use in humans, renders the STING agonist GCV a promising candidate in translational development for prophylactic treatments against stroke.^28^

GCV might be particularly propitious to drive Type I IFN responses to prevent stroke and viral replication in the context of COVID-19 infection where IFN countermeasures are inhibited.^40,41^ In cell culture systems, the STING agonist dimeric amidobenzimidazole (diABZI) has been shown to demonstrate anti-coronavirus activity against HCoV-229E and SARS-CoV-2.^42^ DiABZI’s antiviral activity depends on TBK1-IRF activation and the type I IFN response.^42^ GCV thus may diminish the increased risk of stroke and long COVID-19 syndrome seen in patients infected with SARS-COV2.

## Abbreviations

BX795 =: N-[3-[[5-iodo-4-[[3-[(2-thienylcarbonyl)amino]propyl]amino]-2-pyrimidinyl]amino]phenyl]-1-Pyrrolidinecarboxamide hydrochlodride
cGAS =: cyclic GMP–AMP synthase
CMA =: 10-carboxymethyl-9-acridanone
*Cxcl10* =: C-X-C motif chemokine ligand 10
DiABZI =: amidobenzimidazole
DMSO =: dimethyl sulfoxide
DMXAA =: 5,6-dimethylxanthenone-4-acetic acid
EAE =: experimental autoimmune encephalomyelitis
ER =: endoplasmic reticulum
GCV =: ganciclovir
HCoV-229E =: human coronavirus 229E
*Ifit1* =: interferon-induced protein with tetratricopeptide repeats 1
*Ifnβ1* =: interferon beta 1
IREs =: interferon response elements
*Irf3* =: interferon regulatory factor 3
IRF-3 =: interferon regulatory protein-3
*Irf7* =: Interferon regulatory factor 7
KO =: knock out
LDH =: lactate dehydrogenase
LPS =: lipopolysaccharide
MCAO =: middle cerebral artery occlusion
MK801 =: (5*S*,10*R*)-(+)-5-Methyl-10,11-dihydro-5*H*-dibenzo[*a*, *d*]cyclohepten-5,10-imine maleate
*Mx1* =: MX Dynamin Like GTPase 1
NMDA =: N-methyl-D-aspartate
OGD =: oxygen and glucose deprivation
PEG =: polyethylene glycol
poly I:C =: polyinosinic:polycytidylic acid
qPCR =: quantitative polymerase chain reaction
SARS-CoV-2 =: severe acute respiratory syndrome coronavirus 2
STING =: *st*imulator of *in*terferon *g*enes
TBK1 =: tank-binding kinase 1
TLR3 =: Toll-like receptor 3
TLR4 =: Toll-like receptor 4
TLRs =: Toll-like receptors
TTC =: 2,3,5-triphenylterazolium chloride
